# Plasma cathepsin D correlates with histological classifications of fatty liver disease in adults and responds to intervention

**DOI:** 10.1038/srep38278

**Published:** 2016-12-06

**Authors:** Sofie M. A. Walenbergh, Tom Houben, Sander S. Rensen, Veerle Bieghs, Tim Hendrikx, Patrick J. van Gorp, Yvonne Oligschlaeger, Mike L. J. Jeurissen, Marion J. J. Gijbels, Wim A. Buurman, Anita C. E. Vreugdenhil, Jan Willem M. Greve, Jogchum Plat, Marten H. Hofker, Satish Kalhan, Jussi Pihlajamäki, Patrick Lindsey, Ger H. Koek, Ronit Shiri-Sverdlov

**Affiliations:** 1 Molecular Genetics, General Surgery, Paediatrics, Pathology, Population Genetics, Human Biology, Maastricht University Medical Centre, 6200MD, Maastricht, The Netherlands; 2 Surgery, Atrium Medical Center Parkstad, 6419PC, Heerlen, The Netherlands; 3 Pathology and Medical Biology, Molecular Genetics, Medical Biology Section, University Medical Center Groningen, 9713GZ, Groningen, The Netherlands; 4 Pathobiology, Lerner Research Institute, The Cleveland Clinic Foundation, 9500 Euclid Avenue, Cleveland, USA; 5 Clinical Nutrition, University of Eastern Finland, FI-70211 Kuopio, Finland; 6 Clinical Nutrition and Obesity Center, Kuopio University Hospital, FI-70211 Kuopio, Finland; 7 Internal Medicine, Division of Gastroenterology and Hepatology, Maastricht University Medical Centre, 6200MD, Maastricht, The Netherlands

## Abstract

Non-alcoholic steatohepatitis (NASH) is characterized by liver lipid accumulation and inflammation. The mechanisms that trigger hepatic inflammation are poorly understood and subsequently, no specific non-invasive markers exist. We previously demonstrated a reduction in the plasma lysosomal enzyme, cathepsin D (CatD), in children with NASH compared to children without NASH. Recent studies have raised the concept that non-alcoholic fatty liver disease (NAFLD) in adults is distinct from children due to a different histological pattern in the liver. Yet, the link between plasma CatD to adult NASH was not examined. In the current manuscript, we investigated whether plasma CatD in adults correlates with NASH development and regression. Biopsies were histologically evaluated for inflammation and NAFLD in three complementary cohorts of adults (total n = 248). CatD and alanine aminotransferase (ALT) were measured in plasma. Opposite to our previous observations with childhood NASH, we observed increased levels of plasma CatD in patients with NASH compared to adults without hepatic inflammation. Furthermore, after surgical intervention, we found a reduction of plasma CatD compared to baseline. Our observations highlight a distinct pathophysiology between NASH in children and adults. The observation that plasma CatD correlated with NASH development and regression is promising for NASH diagnosis.

The current obesity epidemic is paralleled by an increasing prevalence of non-alcoholic steatohepatitis (NASH). NASH is characterized by hepatic lipid accumulation (steatosis) and inflammation. While steatosis itself is generally considered benign and reversible, the presence of inflammation will eventually set the stage for further liver damage, including fibrosis, cirrhosis and liver cancer^1^,^2^. Currently, elevations in plasma alanine transaminase (ALT), a liver enzyme, are the primary clinical abnormality detected in NASH patients. When hepatocellular injury is present, this liver enzyme is released into the circulation. Although ALT is used as a tool for the routine diagnosis of NASH, it lacks specificity and sensitivity to distinguish between NASH and steatosis^3^,^4^. In order to improve non-invasive diagnosis of NASH, it is of clinical relevance to investigate novel underlying mechanisms during NASH pathophysiology. Although several processes have been identified that participate in the development of hepatic inflammation, the actual mechanisms for the inflammatory response remain uncertain.

We previously demonstrated a clear and direct association between hepatic inflammation and lysosomal cholesterol accumulation inside Kupffer cells (KCs) of low-density lipoprotein receptor knockout (*Ldlr−/−*) mice fed a high-fat, high-cholesterol diet^5^,^6^,^7^. In accordance with our observation in mice, cholesterol-containing KCs were also demonstrated recently in livers of NASH patients^8^. The occurrence of lysosomal cholesterol accumulation has been shown to induce disturbances in the lysosomal (enzyme) trafficking pathway^9^,^10^. In line, we previously detected modified levels of the lysosomal enzyme cathepsin D (CatD) in the plasma of children with NASH compared to children without hepatic inflammation^11^.

Recent studies have raised the concept that non-alcoholic fatty liver disease (NAFLD) in children and adults is distinct due to differences in the histological pattern in the liver as well as pathological characteristics of the disease^12^,^13^,^14^,^15^. Thus far, the relationship between plasma CatD and adult NASH has not been explored. Therefore, as a follow-up study from our data in children, we investigated in the current paper whether plasma CatD in adults correlates with the development and regression of NASH.

Here, we observed in three complementary cohorts of adults (average age > 40 years), with an average obese body mass index (BMI) of > 30 kg/m^2^, that plasma CatD is significantly increased along the development of NASH. As the plasma CatD levels in adults with NASH were the opposite of those previously found in children with NASH^11^, we point toward the existence of a potential different pathophysiology between NASH in children and adults. Our data further show that CatD responds to surgical intervention, which underlines the potential clinical relevance and diagnostic value of plasma CatD in the context of NASH.

## Methods

### Human cohorts; Cleveland cohort, Maastricht cohort and the Kuopio cohort

The design of this study includes three adult cohorts. All three cohorts demonstrated a similar adult age group (average age > 40 years) and a similar average obese BMI (average BMI > 30 kg/m^2^). Across the three cohorts, all liver biopsies were histologically classified for NAFLD/NASH according to the criteria of Brunt and/or Kleiner, which includes steatosis, ballooning, lobular inflammation and portal inflammation^16^,^17^.

The Cleveland cohort consists of NASH patients after the detection of abnormal plasma ALT values. The second and third population (the Maastricht and Kuopio cohort, respectively) include obese patients that are unselected for ALT, *i.e.*, biopsies were taken during bariatric surgery regardless of their ALT levels. Patients from these cohorts are considered to have a wide spectrum of fatty liver diseases, including early stages.

### Definition Cleveland cohort

A total of 127 adults (average age > 40 years) with an average obese BMI > 30 kg/m^2^ were included, of which 43 had biopsy-proven NASH. Subjects with NAFLD were recruited from the metabolic clinics of the Cleveland Clinic and MetroHealth Medical Center in Cleveland, OH. Excluding criteria, the procedure of obtaining and scoring of liver biopsies as well as blood sampling of this NASH Cleveland cohort have been described previously^18^,^19^,^20^. See Supplementary Table S1 for the patient characteristics of the Cleveland cohort.

### Definition Maastricht cohort

47 obese adult patients (average BMI > 30 kg/m^2^, average age > 40 years), undergoing bariatric surgery at the Maastricht University Medical Center or at the Atrium Medical Center Parkstad, Heerlen, the Netherlands, were included in the study. Excluding criteria, the procedure of obtaining liver biopsies and blood sampling of this Maastricht obese cohort has been described previously^21^,^22^. See Supplementary Table S2 for the population characteristics of the Maastricht cohort. Liver biopsies were evaluated by a trained pathologist, according to the criteria of Brunt^17^ and Kleiner^20^ and graded for severity from NASH grade 1 (early-stage), to NASH grade 3 (late-stage) (see Supplementary Table S3).

### Definition Kuopio cohort

All patients undergoing obesity surgery in Kuopio University Hospital are recruited into our ongoing study investigating metabolic consequences of obesity surgery (Kuopio Obesity Surgery Study)^23^,^24^. The study group included 74 obese adult subjects (average BMI > 30 kg/m^2^, average age > 40 years), who were accepted for Roux-en-Y gastric bypass (RYGB) operation over the years 2005–2010. See Supplementary Table S4 for the general population characteristics at the start of intervention. Excluding criteria, the procedure of obtaining and scoring of the liver biopsies, and blood sampling of the Kuopio obese cohort has been described previously^23^,^24^,^25^.

### Use of human subjects

With regard to the Cleveland cohort, the methods in this study were carried out in accordance with approved guidelines by the Institutional Review Boards at the Cleveland Clinic and MetroHealth Medical Center and was conducted according to the relevant guidelines of the protocol. For the Maastricht cohort, the study was carried out in accordance with the approved guidelines by the Medical Ethical Committees of both the Maastricht University Medical Centre and the Atrium Medical Centre Parkstad and conducted in accordance with the revised version of the Declaration of Helsinki (October 2008, Seoul). For the Kuopio cohort, the study protocol was carried out in accordance with approved guidelines by the Ethics Committee of the Northern Savo Hospital District (54/2005, 104/2008 and 27/2010), and it was performed according to the Helsinki Declaration. Written informed consent was obtained from all the subjects.

### Human cathepsin D enzyme-linked immunosorbent assay

Plasma samples were diluted and CatD levels were determined by the CatD enzyme-linked immunosorbent assay according to the manufacturers’ protocol (Uscn Life Science Inc, Wuhan, China). The detection limit ranges approximately from 46.88 to 3,000 pg/ml. Coefficients of variation (CV%) for intra- and inter assays are <10 and <12% respectively. CatD measurements were performed blinded to the histology findings of the study participants.

### Immunohistochemistry

#### Bone marrow-derived macrophages

Bone marrow-derived macrophages (BMDMs) were isolated as described previously^26^. Cells were grown in 12-well plates (Greiner Bio-One) on coverslips (Ø 20 mm; Thermo Scientific). BMDMs were washed twice with PBS and fixed with 4% paraformaldehyde for 15 min at room temperature. Fixed BMDMs were stored at 4 °C in PBS-NaN3 (0.03%) or washed three times with PBS−/− (Gibco) and directly used for immunocytochemistry. Subsequently, cells were permeabilized (0.1% Triton X-100 and 0.2% BSA in PBS) for 15 min and blocked (2% BSA-PBS) for 30 min at room temperature. Primary and secondary antibodies (2% BSA-PBS) were incubated for 1 h at room temperature. Coverslips were washed and mounted onto glass slides using DABCO-glycerol medium (Sigma-Aldrich) containing DAPI (1:10,000; Sigma-Aldrich) in order to counterstain nuclei. The following antibodies were used: goat anti-mouse CatD antibody (1:100, AF1029, R&D systems), rabbit anti-mouse lysosome-associated membrane protein-1 (LAMP-1) antibody (1:200, Ab24170, Abcam), donkey anti-goat IgG NorthernLights (NL)557–conjugated antibody (1:200; NL001, R&D systems), goat anti-rabbit Alexa488 (1:200; Invitrogen). Cells were imaged using a Leica TCS SPE confocal laser scanning microscope (Leica Microsystems GmbH) equipped with an air-cooled argon-krypton mixed gas laser, using oil immersion objectives. ImageJ software was used to process and analyze the images.

#### Liver biopsy specimens

Immunohistochemistry for CatD was performed on paraffin-embedded liver sections originating from the Maastricht cohort. Sections were deparaffinized in xylene and ethanol and rehydrated in water. Antigen retrieval was achieved by incubating sections in 10 mM sodium citrate buffer in 0.05% Tween20, pH 6.0, at 95 °C. After cooling down, sections were quenched in hydrogen peroxide (3%) to suppress endogenous peroxidase activity. Prior to incubation with the first antibody, slides were first incubated with 1 × PBS plus an amplification step: 1:5 Avidin D Block solution followed by an incubation step with Biotin Block solution 1:5 in 1 × PBS (Avidin/Biotin Blocking kit, SP-2001, Vector Laboratories, Burlingame, CA, USA). Sections were then incubated overnight at 4 °C with the primary CatD antibody (Cell Signaling Technology, no. 2284, Danvers, MA, USA; [1:50]) dissolved in 1 × PBS containing 0.05% Tween20. As negative controls sections were incubated without primary antibody. Incubation with the second antibody for biotinylated donkey anti-rabbit IgG (Jackson ImmunoResearch Laboratories, West Grove, PA; [1:50]) was carried out in 1 × PBS containing 0.05% Tween20 for 30 min. Afterwards the slides were washed and incubated for an additional amplification step in 1 × PBS + 1:50 Avidin D solution + 1:50 Biotin solution (Vectastain Elite ABC kit, PK-6100, Vector Laboratories, Burlingame, CA, USA). Then, 13-amino-9-ethylcarbazole (AEC) was applied using an AEC Peroxidase Substrate kit (Vector Laboratories, SK-4200, Burlingame, CA, USA) and haematoxylin for nuclear counterstaining. Sections were enclosed with Faramount aqueous mounting medium. Pictures were taken with a Nikon DMX1200 digital camera and ACT-1 version 2.63 software (Nikon Instruments Europe, Amstelveen, the Netherlands).

### Statistical analysis

The data were analysed by performing two-tailed non-paired *t*-tests using GraphPad Prism for comparing the different groups. The data were expressed as mean ± SEM and considered significant at p < 0.05. The diagnostic accuracy of the combination ALT with CatD was calculated as described previously^11^.

## Results

### General population characteristics of the Cleveland cohort

ALT was significantly higher in patients with hepatic inflammation compared to their age- and gender-matched control subjects (p < 0.001) (see Supplementary Table S1). BMI, low-density lipoprotein (LDL), triglycerides and aspartate aminotransferase (AST) were all increased in subjects scored for hepatic inflammation compared to their matched controls. In line, plasma high-density lipoprotein (HDL) and the AST/ALT ratio were reduced in the group with hepatic inflammation.

### Elevated CatD levels in plasma of patients with hepatic inflammation

**Figure 1:**
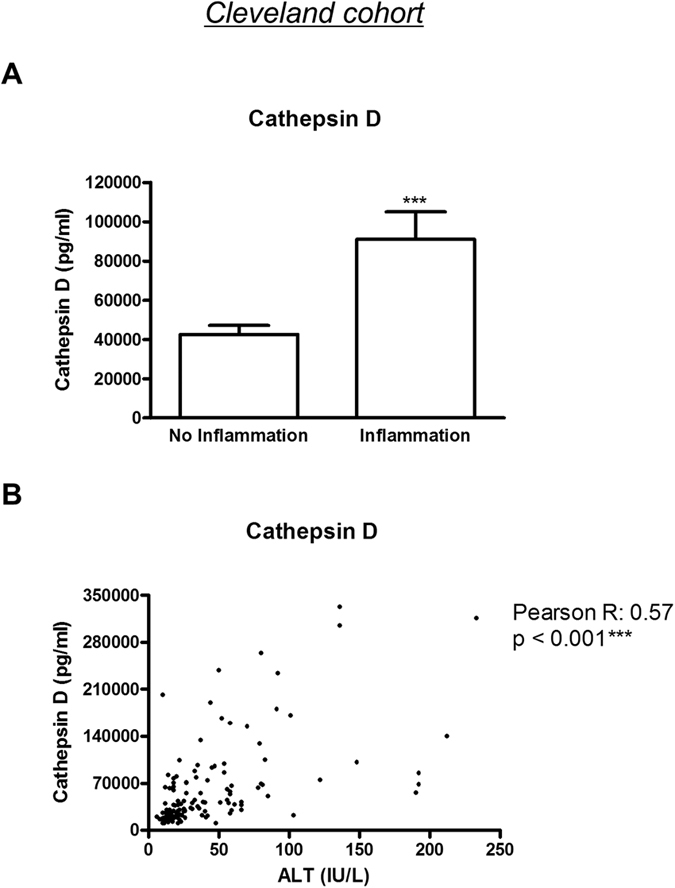
CatD associated with ALT and hepatic inflammation. (**A**) CatD in plasma of subjects with or without hepatic inflammation (n = 127). (**B**) The Pearson correlation test was used to calculate the correlation coefficient (*r*) between plasma CatD and ALT. *** indicates p < 0.001.

First, CatD and ALT were measured in plasma of the Cleveland cohort. We found a significant increase of CatD in relation to liver biopsies of patients that demonstrate inflammation, compared to those without hepatic inflammation (No inflammation *vs.* inflammation: p < 0.001) (Fig. 1A). Next, we investigated the correlation between plasma ALT and CatD. Plasma CatD demonstrated a strong positive correlation with ALT (Pearson’s *r* = 0.57, p < 0.001) (Fig. 1B). These results suggest that plasma CatD levels are linked to the pathogenesis of NASH.

### Population characteristics of the Maastricht cohort

To test whether plasma CatD is elevated already during early-stage of NASH, we used the Maastricht cohort consisting of patients with a wide range of fatty liver disease. Clinical characteristics of the patient population from this cohort are presented in Supplementary Table S2. The BMI from the cohort tested, ranged from 30.7 to 73.6 kg/m^2^ with an overall average of 46.7 kg/m^2^. The average plasma levels of ALT and AST were significantly higher in NASH patients as compared to subjects with a healthy liver (p = 0.018 and p = 0.019 respectively). Patients in the NASH group were significantly older compared to patients with a healthy liver (p = 0.021) and to those who had steatosis (p = 0.049). Importantly, there was no difference with respect to all other parameters (*i.e.*, BMI, plasma total cholesterol, HDL, LDL, triglycerides and the AST/ALT ratio). Liver specimens of 27 NASH patients were histologically classified according to the scoring systems of Brunt and Kleiner. Most NASH subjects (14 out of 27) included displayed mild NASH (grade 1), as summarized in Supplementary Table S3. The majority of NASH patients showed steatosis score 2, ballooning score 1, lobular inflammation score 1, and no or little fibrosis.

### Plasma CatD distinguishes NASH patients from subjects without hepatic inflammation and correlates with histological classifications of NAFLD

**Figure 2:**
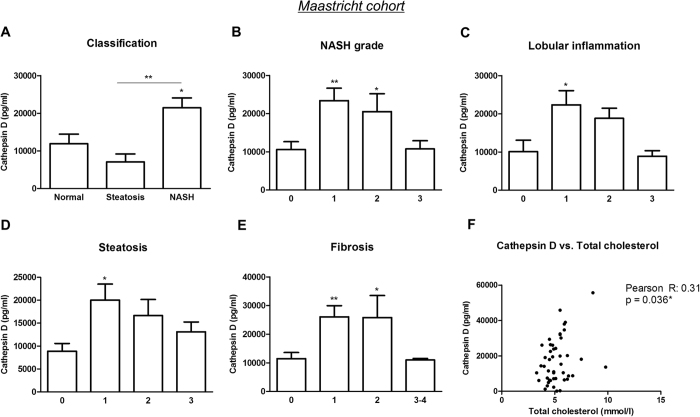
Lysosomal CatD in human plasma. (**A**) CatD in plasma of obese patients with a normal liver phenotype, steatosis and NASH (n = 47). According to Brunt’s criteria, patients were divided for NASH grade (**B**). Lobular inflammation (**C**), steatosis (**D**) and fibrosis (**E**) were categorized according to Kleiner’s scoring system. (**F**) The Pearson correlation test was used to calculate the correlation coefficient (*r*) between plasma CatD and total cholesterol levels. *Significantly different from normal. * and ** indicate p < 0.05, and 0.01, respectively.

The level of plasma CatD was significantly higher in subjects with NASH compared to individuals with either simple steatosis or a healthy liver (NASH *vs*. normal: p = 0.031; NASH *vs*. steatosis: p = 0.0065) (Fig. 2A). More importantly, patients with a mild score for NASH grade, *i.e.*, grade 1, showed an increase in plasma CatD compared to patients without NASH (p = 0.0018) (Fig. 2B). The same significant trend holds true with respect to lobular inflammation, where CatD levels were already increased with mild disease as observed for NASH grade 1 (p = 0.0295) (Fig. 2C). In addition, CatD was strongly elevated upon mild steatosis, *i.e.*, score 1, compared to patients without steatosis (p = 0.019), and decreased upon severity of steatosis (Fig. 2D). Similar to NASH grade, lobular inflammation and steatosis, CatD was primarily elevated upon mild fibrosis (score 1 and 2) compared to patients that did not show fibrosis (p = 0.002 and p = 0.019, respectively) (Fig. 2E). Moreover, total cholesterol levels correlated with plasma CatD (Fig. 2F). In summary, the plasma levels of the lysosomal enzyme CatD are increased in plasma of patients with NASH compared to the patients with a normal liver phenotype or steatosis alone. These data also suggest that plasma CatD correlates specifically with NAFLD severity, already from the early phase, and to cholesterol levels.

### In contrast to ALT, CatD correlates with early stages of NASH

**Figure 3:**
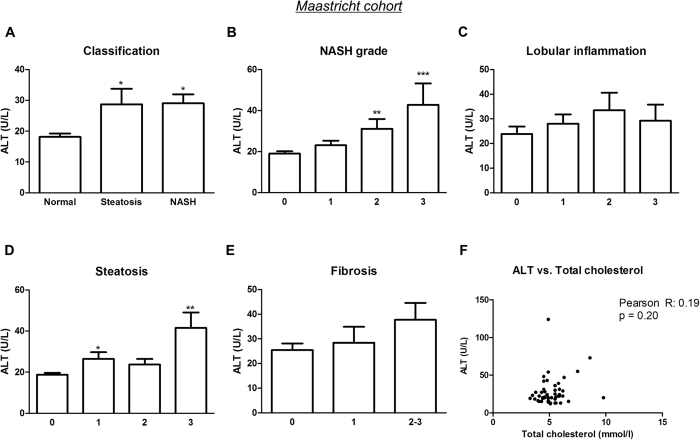
Human plasma ALT levels. (**A**) Plasma ALT in obese patients with either a normal, steatotic or NASH liver (n = 47). Patients were classified for NASH grade (Brunt’s score) (**B**), lobular inflammation (**C**), steatosis (**D**) and fibrosis (**E**) (Kleiner’s score). (**F**) The Pearson correlation test was used to calculate the correlation coefficient (*r*) between plasma ALT and total cholesterol levels *Significantly different from normal. *, ** and *** indicate p < 0.05, 0.01, and 0.001 respectively.

Currently, plasma ALT is the most common diagnostic tool in the clinic to detect hepatocellular injury during NASH. Significantly increased ALT levels were observed in patients with NASH and steatosis (NASH *vs*. normal: p = 0.018; steatosis *vs*. normal: p = 0.019) (Fig. 3A). While plasma CatD could differentiate NASH from steatosis, there was no difference in ALT concentrations between livers with NASH and steatosis, indicating that CatD correlates better with the spectrum of NAFLD than ALT. Moreover, ALT was only increased upon more advanced degrees of hepatic inflammation (NASH grade 2 *vs*. grade 0: p = 0.003; NASH grade 3 *vs*. grade 0: p < 0.001) (Fig. 3B), whereas CatD demonstrated to be elevated already during mild NASH (NASH grade 1). Plasma CatD was significantly higher in the early degrees of steatosis, lobular inflammation and fibrosis, whereas this was not true for ALT (Fig. 3C–E). A correlation was found between CatD and total cholesterol, whereas no similar correlation could be detected for ALT (Fig. 3F). Thus, in contrast to ALT, CatD was elevated upon early NAFLD severity and could distinguish NASH from steatosis in adult subjects with obesity.

### Potential clinical significance of using plasma CatD as an additional non-invasive screening method for NASH

Since our results suggest a strong association between increased plasma CatD and early-stage NASH, we next investigated the potential significance of plasma CatD for the diagnosis of NASH by using the area under the curve (AUC) of plotted receiver-operating characteristic (ROC) curves in the Maastricht cohort. Such a statistical analysis in the Maastricht cohort, with a relative small sample size, gives an indication whether plasma CatD could be potentially clinical relevant for the non-invasive detection of NASH.

#### Normal & Steatosis vs. NASH

**Figure 4:**
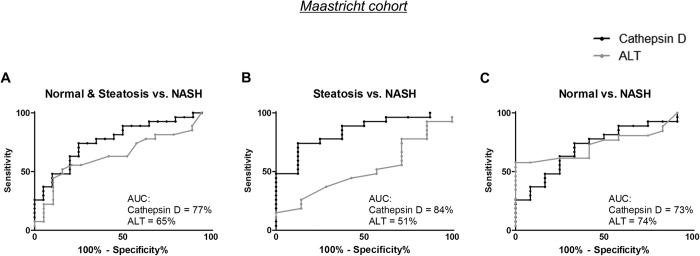
Diagnostic accuracy of ALT and CatD for predicting NASH. ROC curve analysis was used to assess the area under the curve (AUC), the sensitivity and specificity of ALT and CatD in predicting NASH for the following comparisons: healthy & steatotic subjects *vs.* subjects with NASH (**A**); subjects with steatosis *vs*. NASH (**B**); and normal *vs.* NASH subjects (**C**).

First, we compared plasma ALT and CatD levels in normal + steatotic subjects *vs.* patients with NASH. The AUC of ALT alone was estimated to be 65%, whereas CatD displayed an AUC of 77% (Fig. 4A). Notably, combining both enzymes led to an increase in the AUC from 65% to 77% compared to ALT alone (see Supplementary Fig. S1). Thus, the addition of CatD to ALT improved the diagnostic value for distinguishing NASH subjects from those who are healthy or have steatosis.

#### Steatosis vs. NASH

Whereas ALT alone demonstrated low predictive power to discriminate steatosis from NASH (AUC: 51%), CatD demonstrated improved the predictive value with an AUC of 84% (Fig. 4B). In comparison to CatD alone, similar diagnostic values were obtained when ALT and CatD were combined with an AUC value of 84% (see Supplementary Fig. S1). In short, taking plasma CatD into account, the diagnostic prediction for NASH became considerably more accurate than by using ALT alone.

#### Normal vs. NASH

Almost similar AUC values were obtained for ALT and CatD (74% and 73% respectively) for the differentiation between NASH from healthy individuals (Fig. 4C). Combining the two enzymes resulted in a minor increase of the AUC value to 77% (see Supplementary Fig. S1). The relatively high AUC value of ALT alone for the differentiation between NASH and healthy individuals remained roughly equal upon addition of CatD.

### Plasma CatD responds to intervention

**Figure 5:**
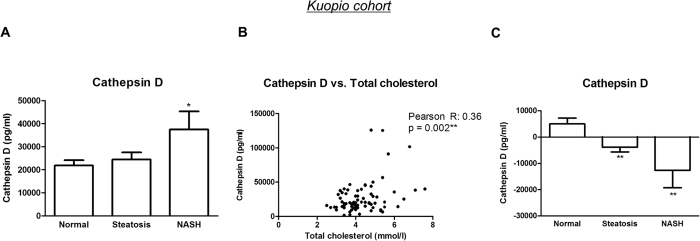
Plasma CatD levels in the Kuopio cohort. (**A**) CatD in plasma of obese patients with a normal liver phenotype, steatosis and NASH (n = 74). (**B**) Correlation between CatD and total cholesterol. (**C**) The absolute difference of plasma CatD was calculated before and after one-year of surgical intervention of normal, steatotic and NASH subjects.

To test the effect of an intervention on plasma CatD levels, we included an additional liver biopsy-proven cohort (*i.e.* the Kuopio cohort), which was monitored following a 1 year surgical intervention. Before the start of the surgical intervention, CatD levels were increased in adults with NASH compared to adults who did not show signs of hepatic inflammation (normal *vs*. NASH: p = 0.039) (Fig. 5A), herewith confirming our previous data in this manuscript (Figs 1A and 2A). Additionally, when merging all three individual cohorts (Cleveland, Maastricht and Kuopio cohort) into one single cohort, plasma CatD levels remained increased in patients with hepatic inflammation compared to those without hepatic inflammation (p = 0.0003) (see Supplementary Fig. S2). Similar to the Maastricht cohort, CatD correlated with total cholesterol levels in plasma (Fig. 5B). The absolute difference of plasma CatD was calculated before and after surgical intervention and demonstrated a significant reduction upon intervention of NASH patients compared to intervention of their healthy individual controls (p = 0.007) (Fig. 5C). These data suggest for the first time that plasma CatD can be used in the clinical follow-up of adult NASH patients.

### CatD localization

Additional to plasma CatD measurements, a CatD staining on normal, steatotic and NASH livers was performed and revealed that CatD expression in liver is similar between the three groups and is mainly located around the pericentral vein (see Supplementary Fig. S3). Next to this, we examined intracellular CatD levels and CatD location in more detail. Bone marrow-derived macrophages (BMDMs) were loaded with oxidized low-density lipoproteins (oxLDL), *i.e.* to mimick hyperlipidemia, or with control medium for 24 h. Our data pointed towards an increase of intracellular CatD upon oxLDL loading, compared to control. Additionally, CatD was located closer to the plasma membrane in oxLDL-treated BMDMs, compared to control treatment (see Supplementary Fig. S4). Additionally, we observed yellow-orange dots in the merged images, which indicates that CatD co-localized with lysosomes. Thus, intracellular CatD expression is increased particularly in lysosomes during high cholesterol conditions, and is located closer to the plasma membrane.

## Discussion

The exact mechanisms leading to NASH remain largely unknown. Diagnosing NASH is of critical importance in order to prevent further progression into NAFLD-related cirrhosis and end-stage liver disease. Therefore, better understanding of the mechanisms that cause progression to NASH is important.

We have recently established a strong association between CatD in pediatric NASH and children without hepatic inflammation^11^. In the current paper, we observed an association between plasma CatD and NASH in three biopsy-proven cohorts of adults. Strikingly, whereas we found a decrease of plasma CatD in NASH children, we here show an increase of CatD in the plasma of adults with NASH. These findings suggest a difference in disease pathology between adult and pediatric NASH. Furthermore, our observations point to plasma CatD as a useful non-invasive tool to improve NASH diagnosis in adults.

**Figure 6:**
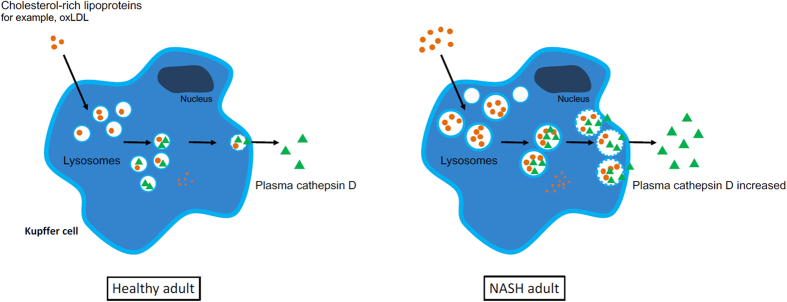
Potential mechanism for enhanced plasma CatD levels during adult NASH. In adults, under normal conditions, circulating cholesterol-rich lipoproteins are endocytosed and directly distributed to the lysosomes. There, lipoprotein-derived cholesteryl esters are further processed following release into the cytoplasm. Under conditions with high cholesterol-rich lipoprotein levels, which is commonly associated with NASH, Kupffer cells internalize high amounts of lipoproteins, leading to enlarged lysosomes. These large cholesterol-filled lysosomes can potentially disturb the trafficking of lysosomes, which can lead to migration of the lysosome towards the plasma membrane. Hence, enhanced extracellular secretion of CatD into the plasma can occur.

Under normal conditions, lipoprotein-derived cholesterol is internalized by macrophages and directed to the lysosome for further processing. After hydrolysis, free cholesterol is transferred from the lysosome to the cytoplasm where the free cholesterol is degraded into bile acid precursors or secreted via cholesterol efflux. The difference in pathophysiology between NASH in children and adults could be due to age-dependent changes of the lysosomal compartment. Indeed, lysosomal function has previously been shown to be an age-dependent process, whereby the amount of lysosomes increase upon ageing^27^,^28^,^29^. These data suggest that at a young age, the amount of lysosomes is low and that, consequently, the cells have difficulties to cope with excessive amounts of cholesterol. As such, upon high-cholesterol circumstances, the stability of the lysosomal membrane is likely to decrease^30^, which can eventually lead to lysosomal rupture and subsequent lysosomal enzyme release into the cytosol. Lysosomal enzyme secretion into the plasma is hereby prevented and instead, the cytosolic lysosomal enzymes activate the apoptotic signaling pathway inducing rapid cell death^31^. This mechanism could explain the reduction of CatD in the plasma of pediatric subjects with NASH compared to subjects without hepatic inflammation^11^. In contrast to young cells, older cells possess a higher number of lysosomes^27^,^28^,^29^, suggesting increased cholesterol storage capabilities inside the cells. It is therefore likely that adult lysosomes can cope better with the cholesterol accumulation and would be less likely to rupture. Nevertheless, cholesterol-filled lysosomes have been shown to induce disturbances in the lysosomal enzyme trafficking pathway^9^,^10^ that can potentially lead to increased levels of lysosomal enzymes in plasma^32^,^33^. Likewise, we observed that CatD is increased intracellularly during high cholesterol conditions and is located closer to the plasma membrane. Since the plasma membrane is the primary site where secretion takes place into plasma^34^, these data suggest that cholesterol-rich lipoproteins, including oxLDL, increases the contact of lysosomes with the plasma and as such leads to elevated levels of plasma CatD. This potential mechanism has been illustrated in a schematic figure (see Fig. 6).

Plasma LDL oxidation can be viewed as a representative parameter of oxidative stress that has been shown to be induced in healthy elderly compared to young controls, and therefore is strongly associated with the course of ageing^35^,^36^. *In vitro,* particularly the intracellular accumulation of the oxidized cholesterol fraction has been shown to enhance extracellular secretion of pro-CatD^37^,^38^. Immunohistochemical stainings of CatD on human liver underlined these findings, as CatD was mainly present in the pericentral area of the liver. Compared to the periportal and the midzonal zones, which are relatively nearer to the entering blood supply, the pericentral zone receives blood that is lower in oxygen and high in oxidative stress^39^. As oxidative stress is increased in NASH patients^40^, these data are in line with our observations in plasma, in which increased levels of CatD are associated with NASH in adults. Altogether, the observed differences between NASH in children and adults could be explained by the fact that secretion of CatD into the plasma is dependent on age-related changes of the lysosomes and therefore is dependent on accumulation of specific lipid species. In agreement, several studies indeed describe that the histological and pathological characteristics of NASH are age-dependent^12^,^13^,^14^,^15^. Further studies are warranted to precisely determine the contribution of lysosomes to NASH disease pathology in children and adults.

With the help of lysosomal enzymes, lysosomes are best known for its primary role in protein degradation. However, protein degradation during NASH, for example of cholesterol, seems to be disturbed, as we have recently established a clear association between hepatic inflammation and increased cholesterol accumulation in lysosomes of KCs^5^. This is underlined by the fact that KCs compromise 15–20% of the total number of liver cells^41^ and account for the main hepatic uptake of modified cholesterol-rich lipoproteins from the circulation^42^. Additionally, numerous studies have shown that lysosomal cholesterol accumulation inside macrophages is an event that occurs during inflammation and has been detected in NASH as well as in atherosclerosis^5^,^6^. These data are in line with previous results showing high cholesterol levels in KCs, as was indicated by numerous cholesterol-filled droplets in KCs of livers of NASH adults, while livers of steatotic patients did not show this typical foam cell-like phenotype^43^.

Lysosomal function is not merely restricted to degradation of proteins, such as cholesterol, but increasing evidence now demonstrates that the lysosomal compartment can also be seen as vesicles that can secrete its content, for example lysosomal enzymes^44^,^45^. Cathepsins, a specific class of lysosomal enzymes, have been shown to be significantly involved in mediating the inflammatory response and cholesterol trafficking^46^,^47^,^48^. Furthermore, we have recently reported that plasma CatD was strongly associated with pediatric NASH^11^. Likewise, a correlation exists between CatD and carotid intima-media thickness, an indicator for atherosclerosis^49^, as well as with a lysosomal storage disease^50^, diseases which both are predominantly characterized by chronic systemic inflammation and excessive cholesterol storage inside macrophages. In line, in the current paper, we show that CatD is correlated with histological classifications for NASH as well as with cholesterol. These evidences point to a strong link between CatD and cholesterol-mediated inflammation. Therefore, it seems reasonable that lysosomal cholesterol accumulation in KCs, and its subsequent effect on lysosomal enzyme homeostasis, is a central mechanism in the development of NASH.

Several plasma markers that have been tested as a potential screening method for NASH are in relation to apoptosis (cytokeratin-18 (CK-18) fragments)^51^, inflammation (cytokines and adipokines)^52^ and advanced glycation end products^53^. Numerous studies screening NASH patients for individual CK-18 fragments described a high diagnostic accuracy with an AUC between 71 and 93%^54^. Our observations for analyzing CatD individually to distinguish NASH from steatotic patients are within the same high accuracy range as CK-18 with an AUC of 84%. However, whereas CatD correlated with early signs of inflammation, CK-18 represents liver damage and fibrosis^51^. Combining two biomarkers such as total CK-18 with interleukin-6 yielded an AUC of 83% and 84% for the combination adiponectin and total CK-18^55^. In line with this approach, we presented the combination of ALT and CatD with a similar high AUC of 84% for separating NASH from steatotic patients. These data suggest that including one liver-specific parameter (*i.e.*, ALT) could increase the diagnostic accuracy for detecting a liver disease such as NASH. In contrast to ALT, increased CatD levels in the plasma are representative for early-stage hepatic inflammation.

Furthermore, we have shown for the first time that plasma CatD responded to a 1-year follow-up period after a gastric bypass intervention of NASH patients. Although no follow-up liver biopsies were obtained in the current study, gastric bypass intervention has previously been shown to resolve histopathological features of NASH^56^. While plasma CatD responded to the intervention, changes in plasma CK-18 levels following intervention of NASH patients have not been defined so far^57^. Contradictive results have been found concerning plasma transaminases after surgical intervention, while others demonstrated a correlation between histological improvement of NASH and lower ALT and AST after follow-up^58^, some did not find a difference postoperatively^56^. Thus, besides NASH diagnosis, plasma CatD can potentially be used in the clinical follow-up of NASH. Further trials are necessary to validate the response of plasma CatD upon a surgical intervention.

Thus, in the current study, we have found a strong association between plasma CatD and NASH in adults. In contrast to childhood NASH, we now demonstrated elevated CatD levels in plasma of NASH adults. These data suggest the existence of a distinct disease pathology between NASH in children and adults. Plasma CatD holds potential clinical utility as it correlated with histological characteristics of NASH and regression of this disease. Mechanistically, these findings point to the important role of lysosomes in the development of NASH and should be further investigated.

## Additional Information

**How to cite this article**: Walenbergh, S. M. A. *et al*. Plasma cathepsin D correlates with histological classifications of fatty liver disease in adults and responds to intervention. *Sci. Rep.* **6**, 38278; doi: 10.1038/srep38278 (2016).

**Publisher's note:** Springer Nature remains neutral with regard to jurisdictional claims in published maps and institutional affiliations.

## Supplementary Material

Supplementary Information

## References

[b1] Kopec K. L. & Burns D. Nonalcoholic fatty liver disease: a review of the spectrum of disease, diagnosis, and therapy. Nutr Clin Pract 26, 565–576 (2011). 21947639 10.1177/0884533611419668

[b2] Yilmaz Y. Review article: is non-alcoholic fatty liver disease a spectrum, or are steatosis and non-alcoholic steatohepatitis distinct conditions? Aliment Pharmacol Ther 36, 815–823 (2012). 22966992 10.1111/apt.12046

[b3] Wieckowska A., McCullough A. J. & Feldstein A. E. Noninvasive diagnosis and monitoring of nonalcoholic steatohepatitis: present and future. Hepatology 46, 582–589 (2007). 17661414 10.1002/hep.21768

[b4] Limdi J. K. & Hyde G. M. Evaluation of abnormal liver function tests. Postgrad Med J 79, 307–312 (2003). 12840117 10.1136/pmj.79.932.307 PMC1742736

[b5] Bieghs V. . The cholesterol derivative 27-hydroxycholesterol reduces steatohepatitis in mice. Gastroenterology 144, 167–178 e161, doi: 10.1053/j.gastro.2012.09.062 (2013). 23041327

[b6] Bieghs V., Rensen P. C., Hofker M. H. & Shiri-Sverdlov R. NASH and atherosclerosis are two aspects of a shared disease: central role for macrophages. Atherosclerosis 220, 287–293 (2012). 21930273 10.1016/j.atherosclerosis.2011.08.041

[b7] Bieghs V. . Internalization of modified lipids by CD36 and SR-A leads to hepatic inflammation and lysosomal cholesterol storage in Kupffer cells. PloS one 7, e34378 (2012). 22470565 10.1371/journal.pone.0034378 PMC3314620

[b8] Ioannou G. N., Haigh W. G., Thorning D. & Savard C. Hepatic Cholesterol Crystals and Crown-Like Structures Distinguish NASH from Simple Steatosis. J Lipid Res (2013). 10.1194/jlr.M034876 PMC3622327 23417738

[b9] Kornfeld S. Trafficking of lysosomal enzymes in normal and disease states. J Clin Invest 77, 1–6 (1986). 3003148 10.1172/JCI112262 PMC423299

[b10] Shen D. . Lipid storage disorders block lysosomal trafficking by inhibiting a TRP channel and lysosomal calcium release. Nat Commun 3, 731 (2012). 22415822 10.1038/ncomms1735 PMC3347486

[b11] Walenbergh S. M. . Plasma cathepsin d levels: a novel tool to predict pediatric hepatic inflammation. The American journal of gastroenterology 110, 462–470, doi: 10.1038/ajg.2015.29 (2015). 25732418

[b12] Nobili V. Non-alcoholic fatty liver disease in children and adolescents. Clinical biochemistry 47, 720, doi: 10.1016/j.clinbiochem.2014.05.025 (2014). 24854694

[b13] Arata M., Nakajima J., Nishimata S., Nagata T. & Kawashima H. Nonalcoholic steatohepatitis and insulin resistance in children. World journal of diabetes 5, 917–923, doi: 10.4239/wjd.v5.i6.917 (2014). 25512797 PMC4265881

[b14] Giorgio V., Prono F., Graziano F. & Nobili V. Pediatric non alcoholic fatty liver disease: old and new concepts on development, progression, metabolic insight and potential treatment targets. BMC pediatrics 13, 40, doi: 10.1186/1471-2431-13-40 (2013). 23530957 PMC3620555

[b15] Ovchinsky N. & Lavine J. E. A critical appraisal of advances in pediatric nonalcoholic Fatty liver disease. Seminars in liver disease 32, 317–324, doi: 10.1055/s-0032-1329905 (2012). 23397532

[b16] Kleiner D. E. . Design and validation of a histological scoring system for nonalcoholic fatty liver disease. Hepatology 41, 1313–1321 (2005). 15915461 10.1002/hep.20701

[b17] Brunt E. M. Pathology of fatty liver disease. Mod Pathol 20 Suppl 1, S40–48 (2007). 17486051 10.1038/modpathol.3800680

[b18] Kalhan S. C. . Plasma metabolomic profile in nonalcoholic fatty liver disease. Metabolism: clinical and experimental 60, 404–413 (2011). 20423748 10.1016/j.metabol.2010.03.006 PMC2950914

[b19] Kasumov T. . Plasma levels of asymmetric dimethylarginine in patients with biopsy-proven nonalcoholic fatty liver disease. Metabolism: clinical and experimental 60, 776–781 (2011). 20869086 10.1016/j.metabol.2010.07.027 PMC3012158

[b20] Kleiner D. E. . Design and validation of a histological scoring system for nonalcoholic fatty liver disease. Hepatology 41, 1313–1321, doi: 10.1002/hep.20701 (2005). 15915461

[b21] Verdam F. J. . Non-alcoholic steatohepatitis: A non-invasive diagnosis by analysis of exhaled breath. Journal of Hepatology 58, 543–548, doi: 10.1016/j.jhep.2012.10.030 (2013). 23142062

[b22] Rensen S. S. . Activation of the complement system in human nonalcoholic fatty liver disease. Hepatology 50, 1809–1817 (2009). 19821522 10.1002/hep.23228

[b23] Pihlajamaki J. . Cholesterol absorption decreases after Roux-en-Y gastric bypass but not after gastric banding. Metabolism: clinical and experimental 59, 866–872, doi: 10.1016/j.metabol.2009.10.004 (2010). 20015521

[b24] Pihlajamaki J. . Serum interleukin 1 receptor antagonist as an independent marker of non-alcoholic steatohepatitis in humans. Journal of hepatology 56, 663–670, doi: 10.1016/j.jhep.2011.10.005 (2012). 22027586

[b25] Brunt E. M., Janney C. G., Di Bisceglie A. M., Neuschwander-Tetri B. A. & Bacon B. R. Nonalcoholic steatohepatitis: a proposal for grading and staging the histological lesions. The American journal of gastroenterology 94, 2467–2474, doi: 10.1111/j.1572-0241.1999.01377.x (1999). 10484010

[b26] Hendrikx T. . Bone marrow-specific caspase-1/11 deficiency inhibits atherosclerosis development in Ldlr(−/−) mice. FEBS J 282, 2327–2338, doi: 10.1111/febs.13279 (2015). 25817537

[b27] Lynch G. & Bi X. Lysosomes and brain aging in mammals. Neurochemical research 28, 1725–1734 (2003). 14584826 10.1023/a:1026069223763

[b28] Robbins E., Levine E. M. & Eagle H. Morphologic changes accompanying senescence of cultured human diploid cells. The Journal of experimental medicine 131, 1211–1222 (1970). 5419270 10.1084/jem.131.6.1211 PMC2138843

[b29] Cho S. & Hwang E. S. Status of mTOR activity may phenotypically differentiate senescence and quiescence. Molecules and cells 33, 597–604, doi: 10.1007/s10059-012-0042-1 (2012). 22570149 PMC3887751

[b30] Yuan X. M., Li W., Olsson A. G. & Brunk U. T. The toxicity to macrophages of oxidized low-density lipoprotein is mediated through lysosomal damage. Atherosclerosis 133, 153–161 (1997). 9298675 10.1016/s0021-9150(97)00094-4

[b31] Singh R. . Autophagy regulates lipid metabolism. Nature 458, 1131–1135, doi: 10.1038/nature07976 (2009). 19339967 PMC2676208

[b32] Ungewickell A. J. & Majerus P. W. Increased levels of plasma lysosomal enzymes in patients with Lowe syndrome. Proc Natl Acad Sci USA 96, 13342–13344 (1999). 10557322 10.1073/pnas.96.23.13342 PMC23949

[b33] Hultberg B., Isaksson A., Sjoblad S. & Ockerman P. A. Acid hydrolases in serum from patients with lysosomal disorders. Clin Chim Acta 100, 33–38 (1980). 6766092 10.1016/0009-8981(80)90182-5

[b34] Mellman I. & Warren G. The road taken: past and future foundations of membrane traffic. Cell 100, 99–112 (2000). 10647935 10.1016/s0092-8674(00)81687-6

[b35] Bottino D. A. . Relationship between biomarkers of inflammation, oxidative stress and endothelial/microcirculatory function in successful aging versus healthy youth: a transversal study. BMC geriatrics 15, 41, doi: 10.1186/s12877-015-0044-x (2015). 25888078 PMC4393601

[b36] Gradinaru D., Borsa C., Ionescu C. & Prada G. I. Oxidized LDL and NO synthesis-Biomarkers of endothelial dysfunction and ageing. Mechanisms of ageing and development, doi: 10.1016/j.mad.2015.03.003 (2015). 25804383

[b37] Hoppe G., O’Neil J., Hoff H. F. & Sears J. Products of lipid peroxidation induce missorting of the principal lysosomal protease in retinal pigment epithelium. Biochimica et biophysica acta 1689, 33–41, doi: 10.1016/j.bbadis.2004.01.004 (2004). 15158911

[b38] Li W., Yuan X. M., Olsson A. G. & Brunk U. T. Uptake of oxidized LDL by macrophages results in partial lysosomal enzyme inactivation and relocation. Arteriosclerosis, thrombosis, and vascular biology 18, 177–184 (1998). 10.1161/01.atv.18.2.177 9484981

[b39] Braeuning A. . Differential gene expression in periportal and perivenous mouse hepatocytes. FEBS J 273, 5051–5061, doi: 10.1111/j.1742-4658.2006.05503.x (2006). 17054714

[b40] Seki S. . *In situ* detection of lipid peroxidation and oxidative DNA damage in non-alcoholic fatty liver diseases. Journal of hepatology 37, 56–62 (2002). 12076862 10.1016/s0168-8278(02)00073-9

[b41] Kolios G., Valatas V. & Kouroumalis E. Role of Kupffer cells in the pathogenesis of liver disease. World J Gastroenterol 12, 7413–7420 (2006). 17167827 10.3748/wjg.v12.i46.7413 PMC4087584

[b42] Van Berkel T. J., De Rijke Y. B. & Kruijt J. K. Different fate *in vivo* of oxidatively modified low density lipoprotein and acetylated low density lipoprotein in rats. Recognition by various scavenger receptors on Kupffer and endothelial liver cells. J Biol Chem 266, 2282–2289 (1991). 1989982

[b43] Ioannou G. N., Haigh W. G., Thorning D. & Savard C. Hepatic cholesterol crystals and crown-like structures distinguish NASH from simple steatosis. Journal of lipid research 54, 1326–1334, doi: 10.1194/jlr.M034876 (2013). 23417738 PMC3622327

[b44] Bordon Y. Immune regulation: lysosomes at the heart of inflammation. Nature reviews. Immunology 11, 502, doi: 10.1038/nri3035 (2011). 21779035

[b45] Samie M. A. & Xu H. Lysosomal Exocytosis and Lipid Storage Disorders. Journal of lipid research, doi: 10.1194/jlr.R046896 (2014). PMC4031951 24668941

[b46] Hannaford J., Guo H. & Chen X. Involvement of cathepsins B and L in inflammation and cholesterol trafficking protein NPC2 secretion in macrophages. Obesity 21, 1586–1595, doi: 10.1002/oby.20136 (2013). 23666609 PMC6445554

[b47] Decock J. . Cathepsin B, cathepsin H, cathepsin X and cystatin C in sera of patients with early-stage and inflammatory breast cancer. Int J Biol Markers 23, 161–168 (2008). 18949742 10.1177/172460080802300305

[b48] Sukhova G. K. . Deficiency of cathepsin S reduces atherosclerosis in LDL receptor-deficient mice. J Clin Invest 111, 897–906, doi: 10.1172/JCI14915 (2003). 12639996 PMC153760

[b49] Moallem S. A. . Correlation between cathepsin D serum concentration and carotid intima-media thickness in hemodialysis patients. International urology and nephrology 43, 841–848, doi: 10.1007/s11255-010-9729-4 (2011). 20387115

[b50] Amritraj A. . Increased activity and altered subcellular distribution of lysosomal enzymes determine neuronal vulnerability in Niemann-Pick type C1-deficient mice. The American journal of pathology 175, 2540–2556, doi: 10.2353/ajpath.2009.081096 (2009). 19893049 PMC2789601

[b51] Feldstein A. E. . Cytokeratin-18 fragment levels as noninvasive biomarkers for nonalcoholic steatohepatitis: a multicenter validation study. Hepatology 50, 1072–1078 (2009). 19585618 10.1002/hep.23050 PMC2757511

[b52] Krawczyk K. . Adipohormones as prognostric markers in patients with nonalcoholic steatohepatitis (NASH). J Physiol Pharmacol 60 Suppl 3, 71–75 (2009). 19996485

[b53] Yilmaz Y. . Decreased plasma levels of soluble receptor for advanced glycation endproducts (sRAGE) in patients with nonalcoholic fatty liver disease. Clinical biochemistry 42, 802–807 (2009). 19217891 10.1016/j.clinbiochem.2009.02.003

[b54] Adams L. A. & Feldstein A. E. Non-invasive diagnosis of nonalcoholic fatty liver and nonalcoholic steatohepatitis. J Dig Dis 12, 10–16 (2011). 21091933 10.1111/j.1751-2980.2010.00471.x

[b55] Grigorescu M. . A novel pahtophysiological-based panel of biomarkers for the diagnosis of nonalcoholic steatohepatitis. J Physiol Pharmacol 63, 347–353 (2012). 23070083

[b56] Barker K. B. . Non-alcoholic steatohepatitis: effect of Roux-en-Y gastric bypass surgery. The American journal of gastroenterology 101, 368–373, doi: 10.1111/j.1572-0241.2006.00419.x (2006). 16454845

[b57] Dyson J. K., McPherson S. & Anstee Q. M. Non-alcoholic fatty liver disease: non-invasive investigation and risk stratification. Journal of clinical pathology 66, 1033–1045, doi: 10.1136/jclinpath-2013-201620 (2013). 23940130

[b58] Dixon J. B., Bhathal P. S., Hughes N. R. & O’Brien P. E. Nonalcoholic fatty liver disease: Improvement in liver histological analysis with weight loss. Hepatology 39, 1647–1654, doi: 10.1002/hep.20251 (2004). 15185306

