# Supplementary material for: Plasma cathepsin D correlates with histological classifications of fatty liver disease in adults and responds to intervention
Source: Sci Rep. 2016 Dec 6;6:38278. doi: 10.1038/srep38278 (PMC5138820; doi:10.1038/srep38278)
Supplement: Supplementary Information [file srep38278-s1.pdf]

Supplementary Information

Plasma cathepsin D correlates with histological classifications of fatty liver disease in adults and responds to intervention

Sofie MA Walenbergh, Tom Houben, Sander S Rensen, Veerle Bieghs, Tim Hendrikx, Patrick J van Gorp, Yvonne Oligschlaeger, Mike LJ Jeurissen, Marion JJ Gijbels, Wim A Buurman, Anita CE Vreugdenhil, Jan Willem M Greve, Jogchum Plat, Marten H Hofker, Satish Kalhan, Jussi Pihlajamäki, Patrick Lindsey, Ger H Koek, Ronit Shiri-Sverdlov

Supplementary Figure S1

Maastricht cohort

ALT + Cathepsin D

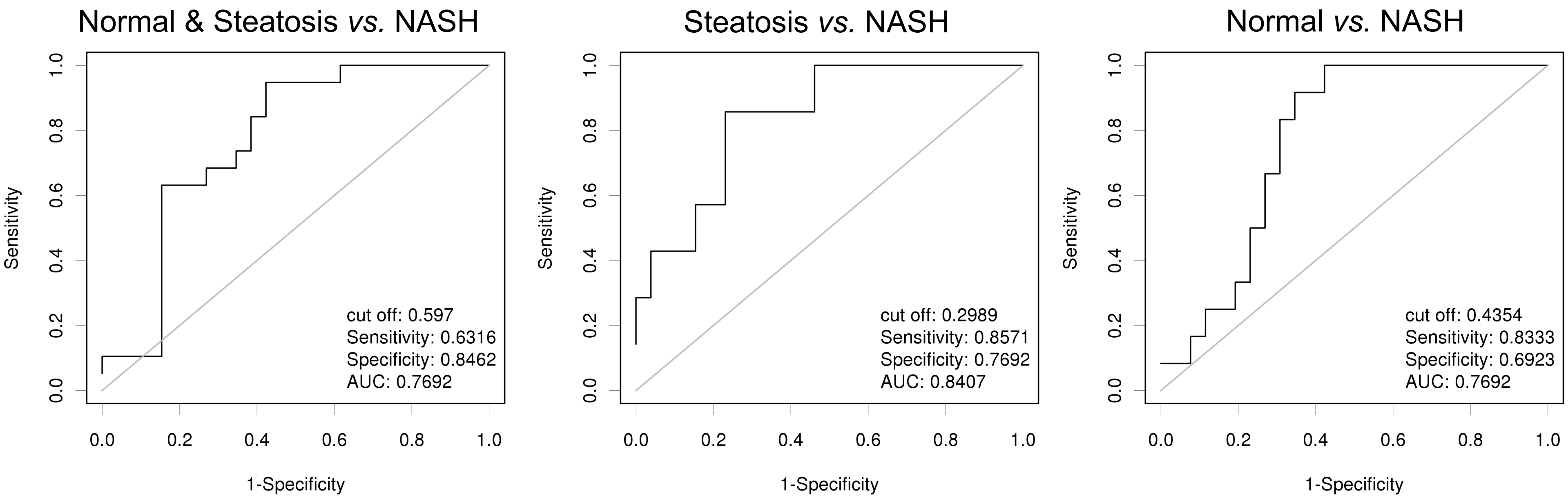

Supplementary Figure S1: Diagnostic accuracy of ALT + CatD combined together to predict NASH.

Supplementary Figure S2

Cleveland cohort + Maastricht cohort + Kuopio cohort

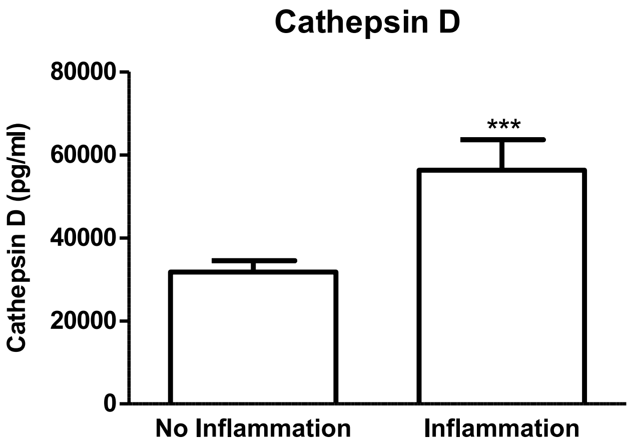

Supplementary Figure S2: Plasma CatD levels in three merged cohorts (Cleveland, Maastricht and Kuopio).

Supplementary Figure S3

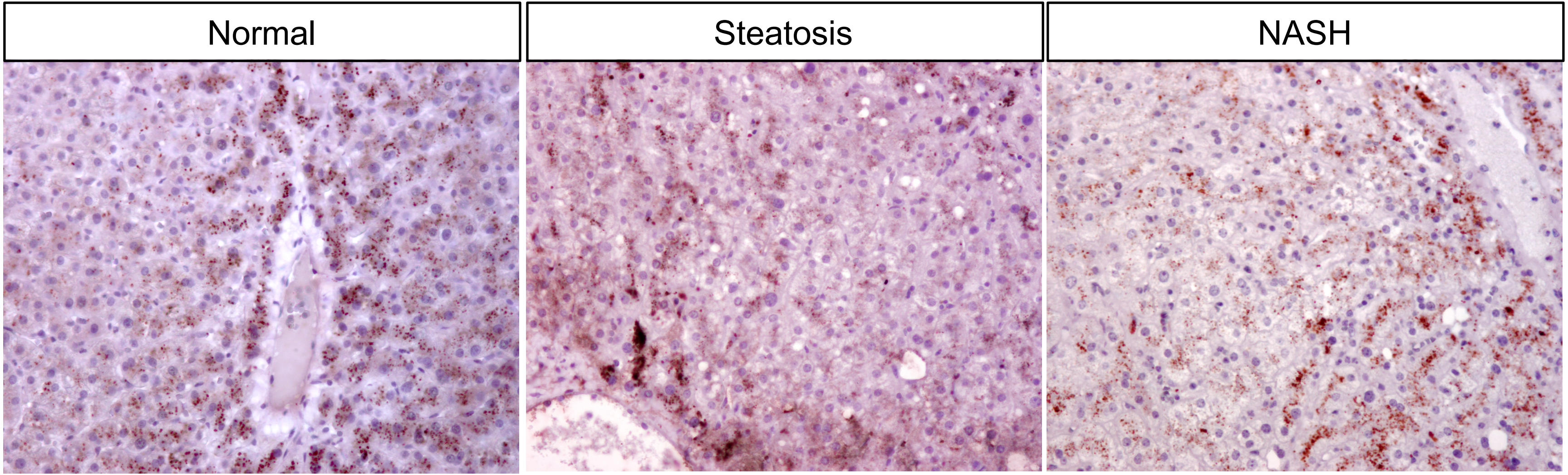

Supplementary Figure S3: Representative pictures of the immunohistochemical staining of CatD on liver biopsy specimens (total n = 5). CatD, which is depicted in red, occurs mainly in the pericentral area of the liver. Magnification 200x.

Supplementary Figure S4

Bone marrow-derived macrophages C57/Bl6

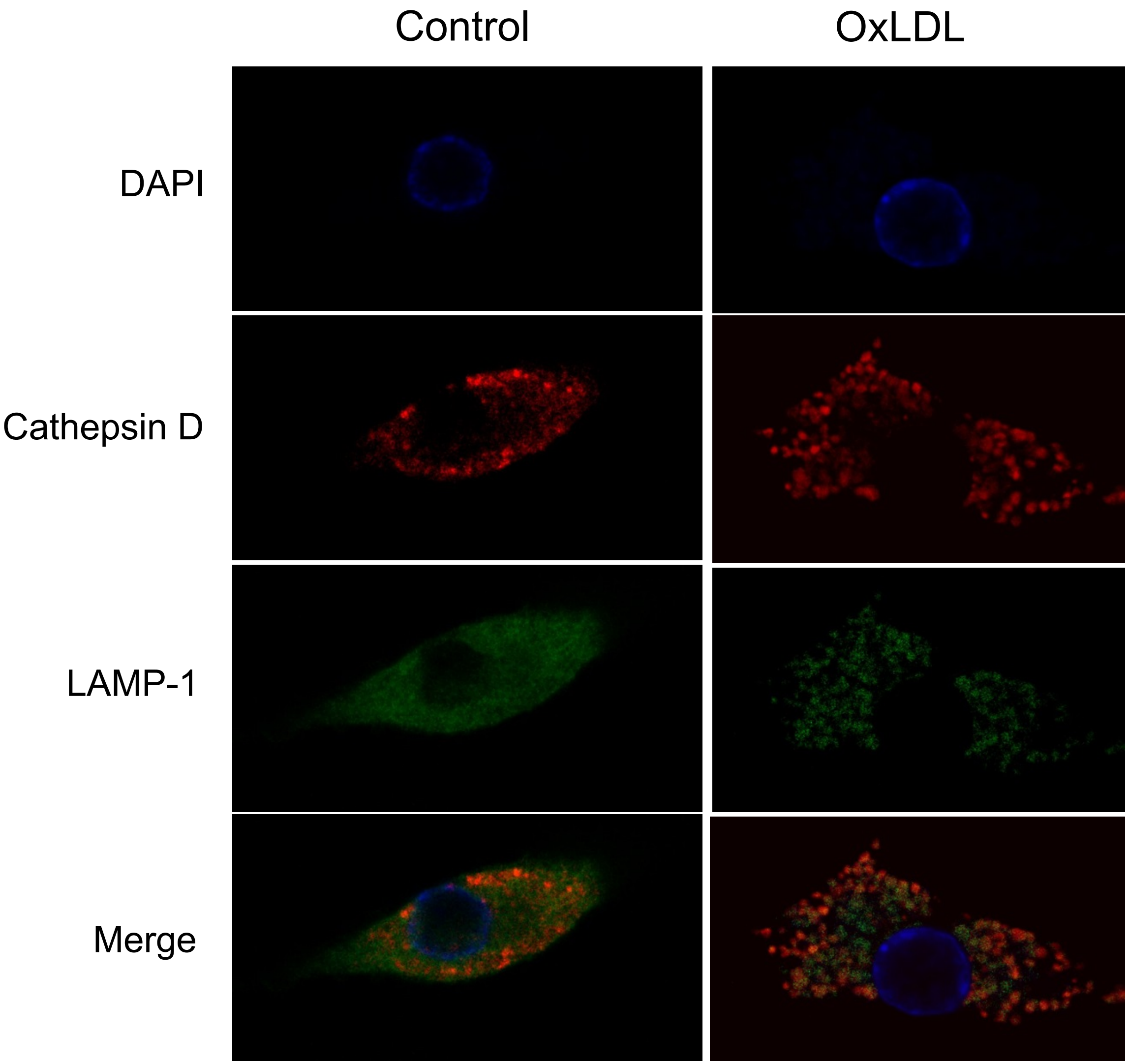

Supplementary Figure S4: CatD expression in bone marrow-derived macrophages (BMDMs) loaded with and without oxidized low-density lipoproteins (oxLDL). Cells were fixed and stained with an anti-cathepsin D antibody and anti-lysosomal-associated membrane protein 1 (LAMP-1) antibody that was detected by a NorthernLights557-labeled (in red) and Alexa488-labeled secondary antibody (in green), respectively. DAPI was used to visualize the nuclei (in blue). Magnification 630x.

Supplementary Table S1. Population characteristics of the Cleveland cohort

	**Cleveland cohort**	
	**No inflammation**	**Inflammation**
n	84	43
Sex, male/female	35/49	17/26
Age, y	41.8±1.3	45.8±1.8
BMI, kg/m^2	28.2±0.7	34.8±0.8***
Total cholesterol, mmol/L	4.7±0.1	5.0±0.2
HDL, mmol/L	1.3±0.1	1.1±0.04*
LDL, mmol/L	2.8±0.1	3.2±0.1*
Triglycerides, mmol/L	1.3±0.1	1.8±0.1*
ALT, IU/L	24.1±2.1	77.0±8.5***
AST, IU/L	24.2±1.0	60.2±6.3***
AST/ALT ratio	1.2±0.1	0.9±0.1***

Data are represented as mean ± SEM.

Significantly different from *No inflammation*, * and *** indicates p<0.05 and p<0.001, respectively.

Supplementary Table S2. Population characteristics of the Maastricht cohort

	Maastricht cohort		
	**Normal**	**Steatosis**	**NASH**
n	12	8	27
Sex, male/female	5/12	0/8	8/19
Age, y	40.5±2.3	40.0±3.9	48.5±2.0*, #
BMI, kg/m^2	43.5±2.0	44.6±2.5	48.8±1.8
Total cholesterol, mmol/L	4.9±0.3	5.4±0.3	5.5±0.3
HDL, mmol/L	0.9±0.1	1.0±0.1	1.0±0.1
LDL, mmol/L	3.1±0.2	3.4±0.4	3.5±0.2
Triglycerides, mmol/L	1.8±0.2	2.2±0.4	2.8±0.6
ALT, IU/L	18.2±1.1	28.7±5.1*	29.0±2.9*
AST, IU/L	18.3±2.2	22.4±2.8	29.5±2.6*
AST/ALT ratio	1.1±0.2	0.8±0.1	1.1±0.1

Data are represented as mean ± SEM. * and # indicate p<0.05. Asteriks (*) indicate significant changes compared with Normal. Hashtag (#) indicates significant changes compared with Steatosis.

Supplementary Table S3. Histological scoring of liver biopsies from NASH subjects of the Maastricht cohort

**Brunt score**	**Definition**	**NASH subjects (n = 27)**
Grade 1	Mild	14
Grade 2	Moderate	7
Grade 3	Severe	6
**Kleiner score**		
Steatosis	<5% (score 0)	0
	5-33% (score 1)	7
	33-66% (score 2)	12
	>66% (score 3)	8
Ballooning	None (score 0)	6
	Few balloon cells (score 1)	18
	Prominent ballooning (score 2)	3
Lobular inflammation	None (score 0)	2
	<2 foci per 200x field (score 1)	14
	2-4 foci per 200x field (score 2)	7
	>4 foci per 200x field (score 3)	4
Fibrosis	None (score 0) / Nondefined	10 / 2
	Perisinusoidal or periportal (score 1)	6
	Perisinusoidal and portal/periportal (score 2)	5
	Bridging fibrosis (score 3)	2
	Extensive bridging fibrosis, cirrhosis (score 4)	2

Supplementary Table S4. Population characteristics of the Kuopio cohort

	**Kuopio cohort**		
	**Normal**	**Steatosis**	**NASH**
n	29	23	22
Sex, male/female	8/21	3/20	10/12
Age, y	50±1.7	46±2.0	47±2.0
BMI, kg/m^2	42.8±1.2	44.6±1.0	44.7±1.3
Total cholesterol, mmol/L	4.1±0.1	4.0±0.2	5.0±0.3**, ##
HDL, mmol/L	1.1±0.05	1.0±0.04	1.1±0.09
LDL, mmol/L	2.3±0.1	2.3±0.2	3.0±0.2**, ##
Triglycerides, mmol/L	1.4±0.1	1.6±0.1	1.9±0.2*
ALT, IU/L	36.9±5.0	42.7±3.8	64.1±10.6*
AST, IU/L	28.6±2.8	27.1±1.7	46.8±8.4 *, #
AST/ALT ratio	0.8±0.07	0.7±0.03	0.8±0.1

Data are represented as mean ± SEM.

Similar to * and **, # and ## indicate $p<0.05$ and $p<0.01$, respectively. Asteriks (*) indicate significant changes compared with Normal. Hashtag (#) indicates significant changes compared with Steatosis.
